# Cystic Neoplasms of the Pancreas: Differential Diagnosis and Radiology Correlation

**DOI:** 10.3389/fonc.2022.860740

**Published:** 2022-03-01

**Authors:** Feixiang Hu, Yue Hu, Dan Wang, Xiaowen Ma, Yali Yue, Wei Tang, Wei Liu, Puye Wu, Weijun Peng, Tong Tong

**Affiliations:** ^1^ Department of Radiology, Fudan University Shanghai Cancer Center, Department of Oncology, Shanghai Medical College, Fudan University, Shanghai, China; ^2^ Hefei Cancer Hospital, Chinese Academy of Sciences (CAS), Hefei, China; ^3^ Shanghai Municipal Hospital of Traditional Chinese Medicine, Shanghai, China; ^4^ Children’s Hospital, Fudan University, Shanghai, China; ^5^ General Electric (GE) Healthcare, Shanghai, China

**Keywords:** pancreatic cystic neoplasms (PCNs), radiology, intraductal papillary mucinous neoplasms (IPMNs), mucinous cystic neoplasms (MCNs), solid pseudopapillary neoplasms (SPN), serous cystic neoplasms (SCN)

## Abstract

Although the probability of pancreatic cystic neoplasms (PCNs) being detected is raising year by year, their differential diagnosis and individualized treatment are still a challenge in clinical work. PCNs are tumors containing cystic components with different biological behaviors, and their clinical manifestations, epidemiology, imaging features, and malignant risks are different. Some are benign [e.g., serous cystic neoplasms (SCNs)], with a barely possible that turning into malignant, while others display a low or higher malignant risk [e.g., solid pseudopapillary neoplasms (SPNs), intraductal papillary mucinous neoplasms (IPMNs), and mucinous cystic neoplasms (MCNs)]. PCN management should concentrate on preventing the progression of malignant tumors while preventing complications caused by unnecessary surgical intervention. Clinically, various advanced imaging equipment are usually combined to obtain a more reliable preoperative diagnosis. The challenge for clinicians and radiologists is how to accurately diagnose PCNs before surgery so that corresponding surgical methods and follow-up strategies can be developed or not, as appropriate. The objective of this review is to sum up the clinical features, imaging findings and management of the most common PCNs according to the classic literature and latest guidelines.

## Introduction

In recent years, the diagnostic rate of PCNs has risen and still keeps an increasing trend. Most patients have no clinical symptoms, and many are found incidentally. Because the etiology and malignant potential of PCNs are often not very clear, diagnosis and management of these neoplasms are challenging. Except for the tail, most of the pancreas is located outside the peritoneum on the posterior wall of the abdominal cavity. Early detection of PCNs is difficult in the absence of clinical symptoms, due to its deep position; most of them are detected incidentally on cross-sectional imaging, and these patients do not have typical pancreatic symptoms (i.e., pancreatitis, jaundice, and new-onset diabetes) (1). It is estimated that approximately 2% to 49% of routine imaging examinations such as computed tomography (CT) or magnetic resonance imaging (MRI) incidentally detect pancreatic cystic lesions, and this proportion increases with age (2).

The most common PCNs are IPMN, MCN, SCN and SPN. Based on epidemiological data, the incidence of MCN, SCN, and SPN in women is significantly higher than that in men, accounting for approximately 95%, 70%, and 80%, respectively, while IPMN is similar or slightly higher than that in men, approximately 55% (3). In recent years, people’s consciousness of these neoplasms has increased, partly due to the application of high-resolution CT/MR imaging. Although the awareness environment of the natural history and most appropriate treatment of various types of cystic lesions is gradually ameliorating, the diagnosis and treatment of PCNs are still challenging. They are usually composed of different solid components, and each solid component exhibits divergent biological behavior, occurring from benign to borderline or even malignant (4). Due to the partial overlap of benign and malignant imaging features, the differential diagnosis of benign versus malignant PCNs is relatively difficult. Moreover, in view of the potential malignant transformation of some benign tumors, the “silent epidemic” of symptomless PCNs has created a real predicament in the treatment and management strategies of such patients.

Different PCNs have relatively specific imaging manifestations, so we can not only identify these tumors by morphological features but also objectively and quantitatively analyze the tumor phenotype by radiomics. Radiomics features have broad application prospects in differential diagnosis, prognosis and efficacy evaluation of PCNs (5). Combined with clinical manifestations and imaging features, radiomics provides an opportunity for the preoperative accurate diagnosis of pancreatic cystic tumors. This review will focus on the clinical features and typical imaging manifestations of different types of PCNs and discusses the latest radiomics research. Thus, it provides an important reference for the precise preoperative diagnosis and individualized management of PCNs. A comprehensive discussion of nonneoplastic pancreatic cystic lesions (PCLs) are not included in this review.

## The Major Pancreatic Cystic Neoplasms

**Figure 1:**
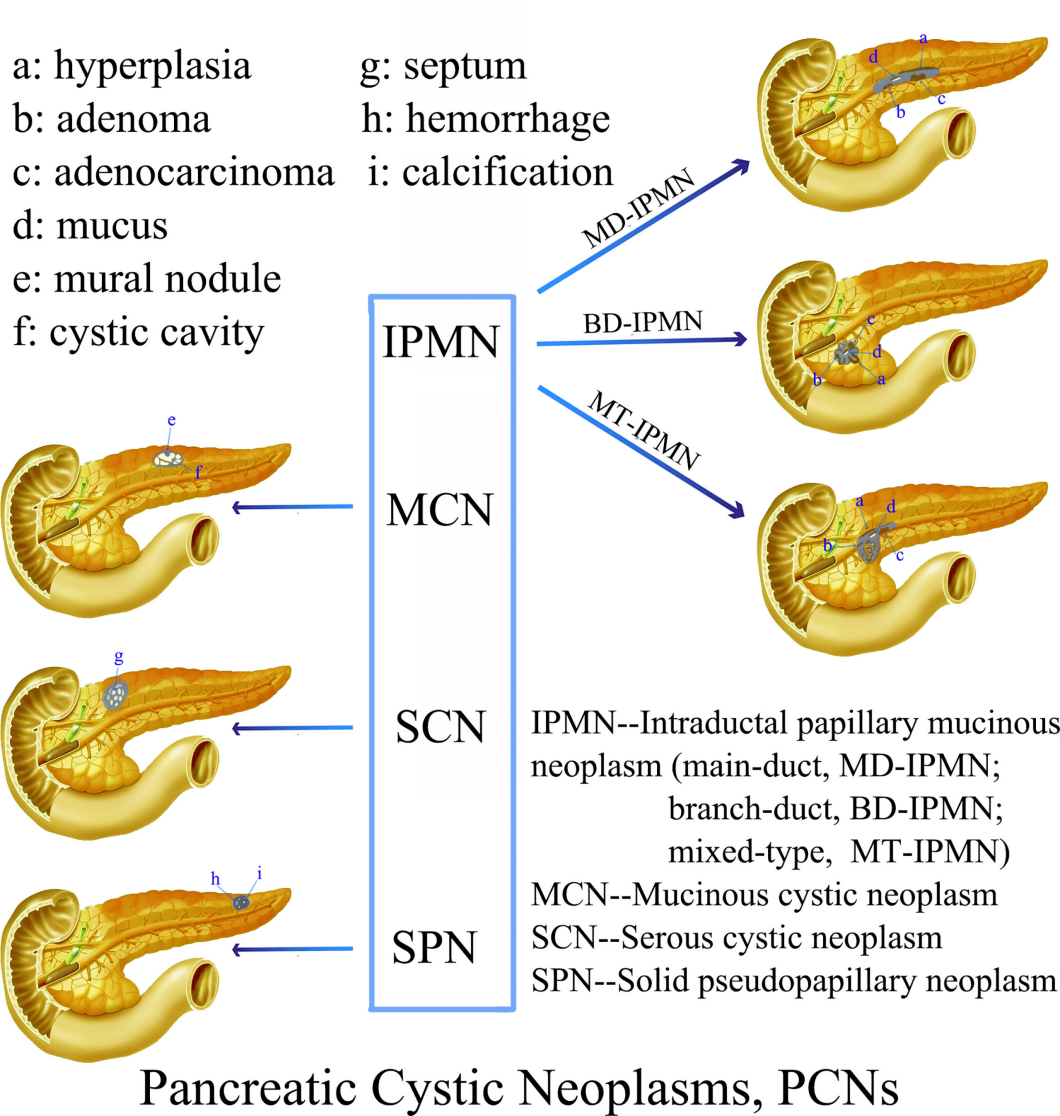
Schematic representation of the characteristic morphological and imaging features of various PCNs.

The most common types of PCNs (**Figure 1**) are mucin-producing intraductal papillary mucinous neoplasms (IPMNs, encompassing branch-duct IPMNs, main-duct IPMNs and mixed-type IPMNs) and mucinous cystic neoplasms (MCNs). Less common subtypes include nonmucinous tumors such as solid pseudopapillary neoplasms (SPNs) and serous cystic neoplasms (SCNs) (6). The incidence of PCNs varies with the population distribution. For example, IPMNs approximately 21% to 33%, MCNs account for 10% to 45%, SPNs account for less than 10%, and SCNs account for 32% to 39% of all PCNs in the Western Hemisphere. There was a national survey report from Korea shows that IPMNs approximately 41%, MCNs approximately 25.2%, SPNs approximately 18.3%, SCNs approximately 15.2%, and others account for 0.3% of PCNs (7, 8). However, the actual incidence of various types of PCN is unknown. The estimated relative frequencies of PCNs removal from one center of 14,424 patients that treated with surgery over a 15-year period were as detailed below: MD-IPMN, BD-IPMN, MCN, SCA, and SPN account for 25%, 26%,11-18%,13-23% and 4-7%, respectively (9). PCNs often do not have typical clinical symptoms. An Italian multicenter prospective study of pancreatic cystic tumors showed that 338 of 1370 cases (24.7%) had one or more clinical symptoms: abdominal pain (214, 15.6%), acute pancreatitis (106, 7.7%), diarrhea (12, 0.9%), gallstones (39, 2.8%), weight loss (21, 1.5%), fatigue (9, 0.7%), loss of appetite (2, 0.1%), diabetes (40, 2.9%) and jaundice (14,10%). However, most patients were asymptomatic (1036, 75.6%) (10). The specific characteristics of the four most common PCNs are exhibition in **Table 1**.

**Table 1:** The clinical and image features of common pancreatic cystic neoplasms (4, 10–27).

Characteristics	IPMN	MCN	SCN	SPN
Age (decade)	5th-7th	4th-5th	5th-6th	2-3th
Gender distribution	Equal	90-95% female	70% female	90% female
Location	Common in pancreatic head	Body and tail	Entire pancreas	Throughout, common in body and tail
Imaging features	Multiple mural nodules, pancreatic duct dilatation, ductal communication, cyst or cluster of cysts	Large cysts with thick septae, peripheral calcification, mural nodules	Star-shaped central scar with calcification, microcystic multiple small cyst, sometimes oligocytic	Local capsule interruption, cystic degeneration, calcification and hemorrhage, floating cloud sign
Cyst fluid	Viscous, mucin-rich	Viscous, mucin-rich	Thin	Bloody
Classification	MD-IPMN, BD-IPMN, MT-IPMN	Low- or intermediate-grade dysplasia, high-grade dysplasia, an associated invasive carcinoma	SMA, SOA, SSCA	Low malignant neoplasm
Clinical symptoms	Incidental finding, jaundice, pancreatitis, malignancy-related	Incidental finding, abdominal pain, malignancy-related	Incidental finding, abdominal pain, mass effect	Incidental finding, abdominal pain, mass effect
Connection with MPD	Yes	No	No	No
Solitary or multifocal	Solitary/multifocal	Solitary	Solitary	Solitary
CEA	≥192~200ng/ml (80%)	≥192~200ng/ml (80%)	<5ng/ml	Unknown
Amylase	High	Low	Low	Low
Molecular markers	KRAS mutation(+) (80%), GNAS mutation(+) (41-66%)	KRAS mutation(+) (50%-75%), GNAS mutation(-), CTNNB1 mutation(-)	VHL, VEGF-A>8500pg/mL, VEGF-C>200pg/mL, MUC1, MUC6	CTNNB1, B-catenin, LEF1, TFE3S, SOX11
Cytology	Columnar cells, +mucin, variable atypia	Columnar cells, +mucin, variable atypia	Often acellular or cuboidal cells stain, +glycogen	Branching papillae with myxoid stroma
Malignant probability	Medium or high	Medium	Negligible	Low or medium

IPMN, intraductal papillary mucinous neoplasms; MCN, Mucinous Cystic Neoplasm; SCN, Serous Cystic Neoplasm; SPN, solid pseudopapillary neoplasms; MPD, main pancreatic duct; MD-IPMN, main-duct IPMN; BD-IPMN, branch-duct IPMN; MT-IPMN, mixed-type IPMN; SMA, serous microcystic adenoma; SOA, serous oligocystic adenoma; SSCA, solid serous cystadenomas.

### Intraductal Papillary Mucinous Neoplasm

IPMN is a benign, borderline, low-grade dysplasia or invasive cancer derived from pancreatic ductal epithelium. Tumor cells are tall columnar mucous-rich epithelial cells, with or without papillary protrusions, extensively invading the main pancreatic duct (MPD) and/or branch pancreatic duct (BPD), causing cystic dilation. With the continuous development of diagnostic standardization and imaging techniques, IPMNs are becoming increasingly routinely diagnosed in clinical practice. According to the communication with the pancreatic duct, they are morphologically divided into MD-IPMN, BD-IPMN and MT-IPMN. Approximately 40% to 65% of IPMNs occur in the branch pancreatic ducts, while they are found in the MPD accounting for about 15% to 35% cases. The probability of simultaneous occurrence in the both pancreatic ducts is only 15%-20% (28).

According to the different histology and mucin expression of IPMN, it can be divided into four epithelial subtypes as below: gastric-type, intestinal-type, pancreatobiliary-type and oncocytic-type, each of which has various kinds of risks of malignant progression. Oncocytic- and gastric-type IPMNs are often low-grade neoplasms, while intestinal- and pancreatobiliary-type IPMNs have a disposition to high-grade neoplasms and are usually related to invasive cancer (IC) (11). The prognosis of IPMNs is superior to pancreatic ductal adenocarcinoma (PDAC) after surgical resection (29). It has been reported that the incidence of cancers derived from IPMN is between 6% and 46% (30–33), including IPMN with high-grade dysplasia (HGD) and IC, and its prognosis is as poor as that of PDAC (34). Assessing the rate of malignancy in IPMN has become a clinical challenge. The risk factors for malignant tumors include weight loss, patient age, relationship with mural nodules, increased jaundice/bilirubin levels, and elevated CEA levels. However, there is no established standard that can safely and accurately distinguish malignant and nonmalignant lesions (35). Therefore, the key to the treatment of IPMN is to accurately predict the risk of malignancy. At the same time, it is also important to evaluate the probability of surgical resection benefit. Imaging takes a significant role in the evaluation and detection of IPMNs (36).

The aims of imaging examination of IPMN are as follows: first, to detect IPMN and exclude other PCLs; second, to distinguish the relationship between lesions and the pancreatic duct, which is conducive to typing; and third, to determine the key risk factor of malignancy and estimate the resectability of clinical surgery. Various imaging methods are used to achieve these goals.

### Mucinous Cystic Neoplasm

MCNs are cystic tumors derived from the pancreatic epithelium that have the potential for malignancy. They are relatively rare pancreatic cystic tumors in the clinic, accounting for 29% of all PCNs (37). Compared with IPMNs, MCNs do neither communication with the MPD nor BPD system. They are often solitary and are covered by a thin fibrous cyst wall. The cyst is lined with tall columnar mucous cells that secrete mucin and often form papillae, and the subepithelial stroma is often ovarian-like stroma with abundant cells (38). There is no significant difference in incidence between sexes in their 60s and 70s, and the probability of occurrence in the body/tail of the pancreas is greater than that in the pancreatic head (67.3%–99.4%) (39), with 89.5% in the present series.

The malignant probability of MCNs varies between 6% and 36%, which is still significant (12). The features predictive of malignancy include irregular or focal thickening of the cyst wall, a large volume, and solid content inside or outside the cyst (40, 41). The size (> 8.5 cm) and volume of the MCNs on CT/MR imaging are the only features associated with HGD/IC, and the average growth rate is very slow, about 4 millimeters (0.16 in) every year (42). The mucinous transitional epithelium is the origin of almost whole malignancies arising from MCNs. MCNs can be divided into three major categories according to the grade of dysplasia as well as IPMNs: low- or intermediate-GD, HGD and IC (13, 43). Resection is advocated for whole types of MCNs according to current the guidelines and clinical consensus unless there are contraindications to surgery (44). For MCNs with IC, the prognosis is closely related to the extent of lesions invasion, tumor stage and R0 resection rate. The two- and five-years survival rates of resectable MCNs with IC are 67% and 50%, respectively (7). Therefore, early detection and identification of MCNs with invasive cancer by imaging methods are of great significance.

### Solid Pseudopapillary Neoplasm

SPNs are an rare pancreatic tumor and, as their name implies, have a solid pseudopapillary structure formed by epithelial cells of a single shape in a loose arrangement. They are prone to hemorrhage and cystic transformation. These tumors account for only 0.2~2.7% of all exocrine pancreatic neoplasms (45). According to the morphological and structural characteristics of the lesion, different reports use the nomenclature solid-cystic neoplasm, papillary-cystic neoplasm, solid-cystic acinar neoplasm, solid-papillary neoplasm, but the actual use of “solid pseudopapillary neoplasm” (SPN) is similarly only a descriptive name that represents morphological features yet retains the openness of histogenesis. Regardless of the presence or absence of metastatic disease, SPNs are generally considered to be low-grade tumors with an indolent growth pattern. The origin of these tumor cells in the pancreas is uncertain. There are two classical theories about the origin of SPN: the one suggests that it origins from pluripotent pancreatic cell, then the other proposes a female genital bud origin (46).

Due to the wide application of high-quality and -resolution imaging examinations, mainly US, CT, and MRI, it has been reported high frequency in the past few decades. In a recent review of all SPN description that published in the English journal up to 2014, Law et al. (47) confirmed a total of 2744 cases of this neoplasm. Yao et al. systematically reviewed 2,450 SPN cases in a Chinese population before January 2020, which was published in both the Chinese and English literature (48). They concluded that SPN is an indolent neoplasm and seldom seen that mainly occurs in young females. The clinical manifestations are abdominal masses and abdominal pain, most of them present as pancreatic head and tail space occupying, and the prognosis is excellent after complete resection. Generally, they are indolent, but a few have malignant potential. Regrettably, the prognostic factors that predict malignant potential have been hard to identify (49). Most patients present with local lesions, and only 9-15% have metastases or local infiltration.

At present, the main treatment is still surgical resection, and its prognosis is different from that of pancreatic cancer. According to reports, the five-year survival rate can be as high as 94-97% (45, 50). Rare SPNs can occur at any age and in both genders, especially young females. Although the survival rate is typically high, histological images cannot accurately predict its biological behavior. Lesions without obvious malignant signs but only suspicious morphological signs can also cause late recurrence, metastasis and even death. The exact histogenesis is still unclear, and it may originate from primordial cells. More research on SPT is needs for further clarification.

### Serous Cystic Neoplasms

SCNs are benign tumors of the pancreatic exocrine glands that account for 16%~33.3% of whole cystic neoplasms of the pancreas. It is a slow-growing benign lesion with an extremely low probability of malignant transformation (14, 15, 51). The concept of SCNs as benign disease entities without the risk of malignant transformation was revised after George et al. revealed the first case of a malignant pancreatic SCN in 1989 (52). The malignancy of SCNs, serous cystadenocarcinomas, are limited to 25~30 cases report in the global literature, representing <1% of all SCNs (15), including the largest sample size, which found three patients with serous cystadenocarcinomas among 2622 cases (53). Therefore, SCNs of the pancreas have extremely low malignant potential but are not totally benign.

Patients are often discovered with SCN in their late 50th or early 60th. SCN usually develops in the body/tail of the pancreas. Despite these neoplasms are mostly benign, they often grow slowly and may have large diameters (13). SCNs are representatively honeycombed microcystic tumors consisting of uniform, cuboidal, glycogen-rich epithelial cells. Thus far, there are four known variants of serous cystadenoma, namely, macrocystic serous cystadenoma, solid serous adenoma, VHL-related SCN, and mixed serous neuroendocrine neoplasm, in which the serous epithelial components of these variants are identical to those of serous cystadenoma.

Pancreatic serous cystadenomas are benign lesions and could be regulated by surveillance, which does not commonly mandate surgical resection unless they exhibit aggressiveness or unspecific characteristics that hinder accurate diagnosis. CT is the preferred first-line examination modality for characterizing SCNs and differentiating them from their mimickers (54).

## Imaging Diagnosis and Precision Imaging

Multiple imaging modalities can help to further distinguish a PCN, facilitating the findings, characterization, and evaluation of the presence of aggressive behavior and the evaluation of resectability in patients with obviously malignancy. Imaging modalities have unique advantages and potential weaknesses in terms of PCN evaluation. Radiomics is an emerging field in quantitative imaging that uses techniques that advance imaging features to objectively and quantitatively investigate tumor phenotypes. Noninvasive medical imaging such as US, MRI, CT, and positron emission tomography (PET) can be used to assess tumor and anatomical tissue features for tumor management (55–57). Radiomics can obtain high-content information through identification, extraction, quantitation, and processing to identify imaging signatures or phenotypes.

Information from surrogate imaging biomarkers combined with multifarious demographic, biologic (“omic”) and outcome-driven methods can be used to develop precision medicine strategies. Accurate imaging biomarkers have been described in a large number of neoplasms. Medical images store more information than trained physicians can see; thus, more details about the region of interest that are embedded in this hidden information can be extracted and analyzed by computational tools than has been previously observed (58, 59). Manual identification of cyst type has an accuracy of only 60~70%, even by well-trained radiologists (60). Therefore, the development of imaging markers using radiomics could increase the correct identification of the type and malignant rates of PCNs.

Regrettably, radiological research to assess the risk of PCN malignancies, especially IPMN, is very limited. In one of the earliest research, an algorithm that distinguishes between the four most common types of PCNs (IPMN, MCN, SCA, and SPN) was proposed by Dmitriev and his colleagues. They revealed an integrated model that combines patient demographic factors with intensity and shape characteristics extracted from cyst images. Segmentation of the cystic neoplasms was acquired by a semi-automated graph-based segmentation technique, at the same time, an random forest classifier and convolutional neural networks were applied for feature selection. This groundbreaking research acquired an accuracy of approximately 84% in distinguishing various types of cysts (61). A recent research revealed a computer-aided diagnosis (CAD) scheme based on radiomics and emphasized the role of radiomics analysis as a new noninvasive tool to improve the accuracy of the preoperative diagnosis of SCN (59). Another study showed that a comprehensive nomogram combining clinical characteristics and fusion radiomics features could identify SCNs from mucin-producing PCNs (58). With the in-depth study of radiomics methods in the field of tumors, it is believed that more research will focus on the differential diagnosis of PCNs in the future.

At present, advanced imaging techniques are increasingly utilized in clinical practice, and the detection rate of PCLs has started to increase gradually. For example, Laffan et al. reported that PCLs were detected in about 2.6% of items using multidetector computed tomography (MDCT) (62), suggesting that CT is the first available source of imaging data for diagnosis. One previous study revealed an accuracy of 67-70% for discriminating 130 pancreatic cysts on CT scans, which were performed by two readers with more than 10-years of experience in abdominal diagnosis (60). In addition, the accuracy of MDCT for characterizing PCN ranges from 56% to 85% (63), and the wide availability, high spatial resolution, and rapidity of acquisition make MDCT ideal for the initial PCN assessment (63). Furthermore, the presence of high-risk stigmata, including a solid component or mass within the cyst, or the presence of mural nodules can be identified by CT imaging. However, MDCT also has disadvantages. It is still difficult to characterize the histopathologic subtype of PCN, as their CT features overlap (64). Besides, the ionizing radiation inherent to CT might result in suboptimal use effects, especially for continued follow-up examinations.

The raising availability and use of dual-energy CT scanners may be advantageous to reduce the overall dose to the patient, thus decreasing the number of acquired phases by using virtual unenhanced imaging. Although CT spectral imaging can provide additional information and multiparametric analysis can achieve greater results than single-parameter analysis in differentiating serous and mucinous content, it is difficult to combine multiparametric analysis and CT spectral imaging-derived quantitative parameters to improve the diagnostic performance (65).

Given the extensive use of high-quality and -resolution MDCT, recent studies have assessed advanced computer-based quantitative image analysis to obtain additional information for identifying characteristics that might be helpful to predict high-risk IPMN. Hanania et al. evaluated 53 cases with IPMN and distinguished 14 imaging characteristics (biomarkers) to differentiate between LGD and HGD in IPMN. Using the top 10 of the 14 biomarkers, an AUC of 0.96 was achieved, with a sensitivity of 97% and specificity of 88% (66). The results of this study indicated that HGD/IC IPMNs have distinct radiomics features that could be utilized to stratify patients *via* noninvasive imaging. Permuth et al. also distinguished malignant from benign IPMN by using radiomics with 14 radiologic features in 38 cases; however, they integrated 5-miRNA data and achieved an AUC of 0.92, with a sensitivity of 83% and specificity of 89% (67). Such high sensitivity and specificity are conducive to improving the clinical discrimination ability of benign and malignant IPMNs so that targeted and individualized treatment strategies can be adopted.

Yang et al. (68) identified 25 patients with MCN from 53 patients with SCN using a preliminary model based on texture features (GLCM, GLRLM, GLZLM, and NGLDM) extracted from contrast-enhanced CT images that were selected *via* LASSO regression and random forest classifiers. Fascinatingly, they also found a good correlation among the extracted texture features extracted from CT images of 2 mm and 5 mm thick slices, which had previously been neglected in many previous studies. Although the feature extraction was not affected by a difference in slice thickness, they suggested using CT images with similar slice thicknesses for radiomics analysis. They acquired an accuracy of 74% in the slice thickness of 2 mm group and 83% in the slice thickness of 5 mm group in the validation group.

The proposed radiomics-based CAD scheme could increase the accuracy of the preoperative diagnosis of pancreatic serous cystic neoplasms, showing an AUC of 0.767 in the cross-validation group and 0.837 in the independent validation group (59). It has also been suggested that using CT alone is of limited value in differentiating between serous and mucinous lesions (69). The study showed that PCNs displaying central scarring, central calcification or the circumvascular sign on CT could be diagnosed as SCAs. When either of the first two features is combined with the circumvascular sign, the diagnostic sensitivity could be improved (14). The malignant probability of SCN is significantly lower than that of MCN. Thus, a follow-up observation strategy can be used for some patients.

The reported incidence of detecting asymptomatic pancreatic cysts on MRI is about 15% (70). The prevalence on MRI is higher overall, ranging from 2.4% to 20%, and increases with age to approximately 40% in patients older than 70y (71, 72). T_2_WI are exquisitely sensitive to fluid-filled structures. Thus, small parenchymal pancreatic cysts or the MPD/BPD system with MRCP can be visualized by T_2_-weighted MRI, which is the primary MRI pulse sequence (73). Compared to MDCT, MRI is more sensitive overall for detecting small pancreatic cysts < 3 cm (74). Furthermore, MRI can detect more PCLs smaller than 20 mm than CT. For lesions larger than or equal to 20 mm, MRI can depict a greater level of internal details than CT, which could aid clinicians in making management decisions (75). Hoffman et al. (76) demonstrated that entropy could be prognostic for malignancy by extracting a few intensity histogram-based statistical features from MR images of 18 patients with BD-IPMN. In a concept-of-proof study involving 38 patients, there were 20 benign and 18 malignant IPMNs. MRI/MRCP has an additional advantage over MDCT for patients who require repeated imaging for follow-up because of the lack of radiation exposure. Disadvantages of MRI include its lower spatial resolution, low sensitivity to detect calcifications, and motion-related artifacts.

### The CT and MR Manifestations of IPMN

**Figure 2:**
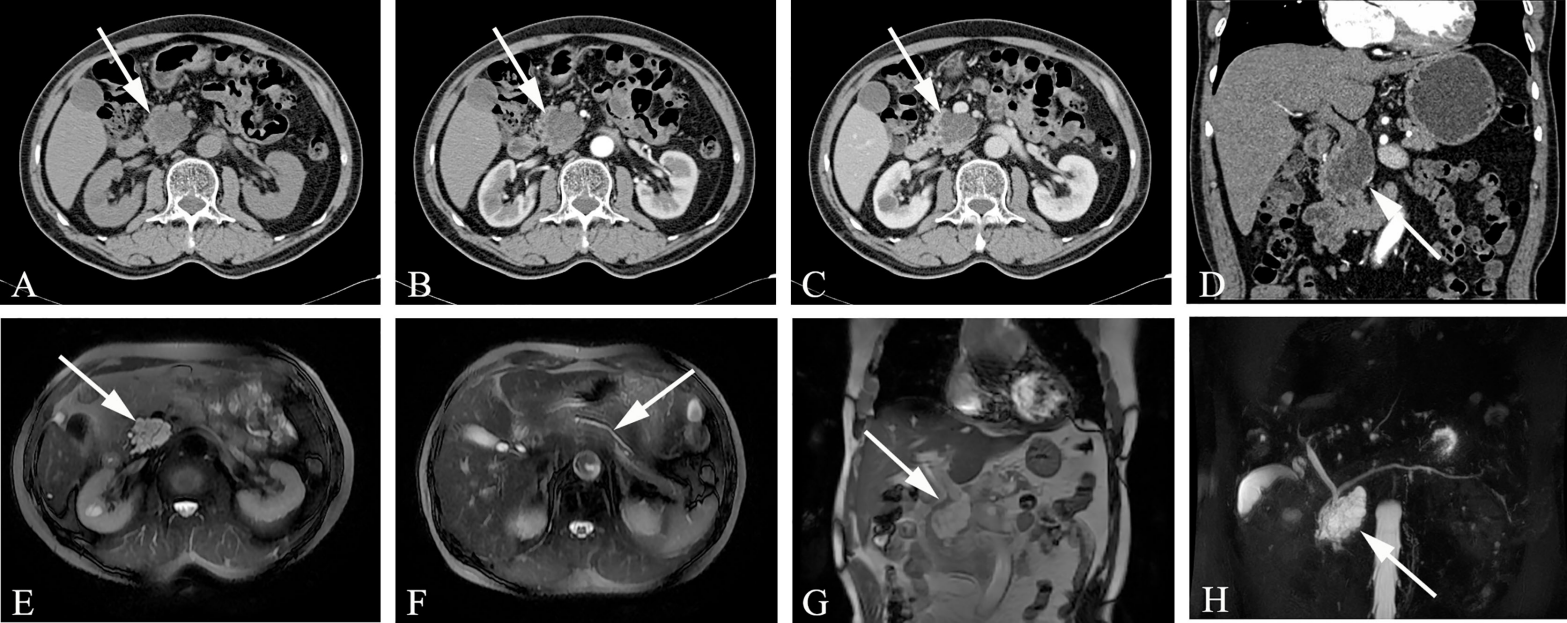
Branch-type IPMN (Male, 66y, physical examination revealed a pancreatic mass for a week). **(A–C)** The CT plain scan, arterial phase and venous phase at the same level; **(D)** Coronal image in arterial phase. **(E)** T_2_-weighted cross-sectional image; **(F)** T_2_-weighted cross-sectional image at another level of the same patient; **(G)**. T_2_-weighted coronal image; H. MRCP reconstruction map; **(A)** The CT plain scan showed multilocular cystic mass of pancreatic head with septation and clear boundary. The density is slightly higher than that of water. **(B–D)** Contrast enhanced scan showed moderate enhancement of mural nodules in the dilated branch pancreatic duct. **(E–H)** Septa can be seen in the lesion and the MPD was slightly dilated. The white arrows in the Figures only indicate the location of the neoplasm.

Among the three types of IPMN, the branched type is the most common, followed by the mixed type, and the MPD type is relatively rare. The latter is further divided into two sub-types, the segmental type and the diffuse type. The communication between cystic lesions and the MPD is one of the diagnostic points of branch-type IPMN. Branch-type IPMN specific imaging findings are as follows: tubular or earthworm-like shadows in low-density cystic lesions, cystic walls and septate microenhancing nodules. The dilated MPD is not limited to the distal end of the lesion. The imaging signs that affect the diagnosis are as follows: the lesions are oval or dumbbell-shaped, the lesions’ density is close to that of water, and there are non-separations or tiny nodules in the lesions. Thin-slice scanning combined with three-phase enhancement, coronal or sagittal image reconstruction, and careful observation of cyst wall and intralesional structures can help improve the diagnostic accuracy (**Figure 2**).

**Figure 3:**
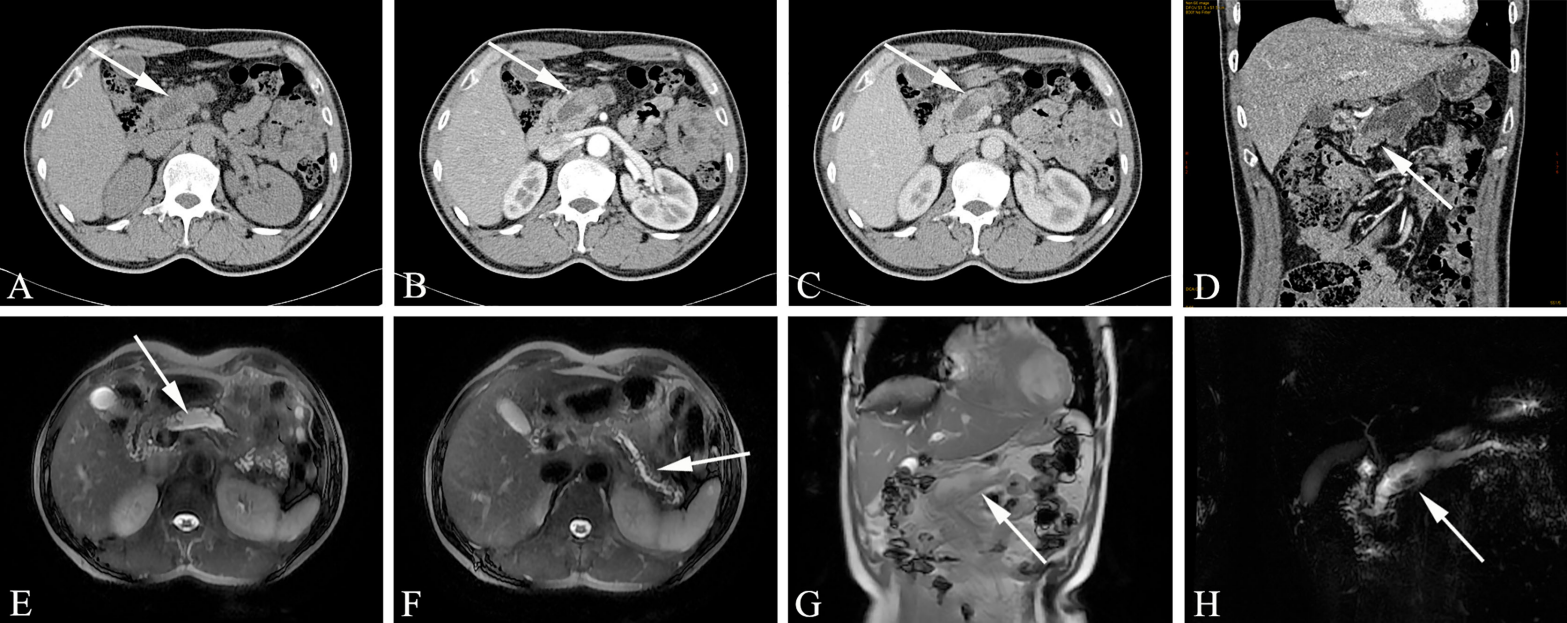
Main-duct IPMN (Male, 57y, abdomen pain for one month). The sequence distribution of images is the same as that in **Figure 2**. **(A)** The CT plain scan showed nodule in the neck of the pancreas, with a CT value of about 31HU. The MPD is obviously dilated, the pancreatic parenchyma is slightly atrophied, the dilated pancreatic duct is low-density, and the density is similar to that of water. **(B, D)** In the arterial phase, the nodule of the pancreatic neck was moderate enhanced, with a CT value of about 65HU, and the dilated pancreatic duct showed more clearly. **(C)** In the venous phase, the nodule showed continuous enhancement, at this time, the CT value is 72HU; **(E–G)** The T_2_-weighted imaging shows pancreatic duct dilatation with multiple mural nodules. **(H)** MRCP shows the MPD dilated significantly throughout the whole pancreas. The white arrows in the Figures only indicate the location of the neoplasm.

The MD-type IPMN-specific imaging manifestations are as below: moderate or greater dilatation of the MPD, continuous expansion of the pancreatic duct without bead-like changes, enhanced mural nodules on the cyst wall, slight atrophy of the pancreatic parenchyma, and markedly dilated MPD asymmetry with mildly atrophied pancreatic parenchyma (**Figure 3**). Imaging signs that affect the diagnosis are as follows: in the early stage of the disease, dilatation of the MPD is limited or mild. In the early stage of the disease, this is easily confused with the slight dilatation of the pancreatic duct caused by anatomical variations; diffuse IPMN is easily confused with chronic pancreatitis, and localized IPMN is easily confused with pancreatic fusiform pseudocysts.

**Figure 4:**
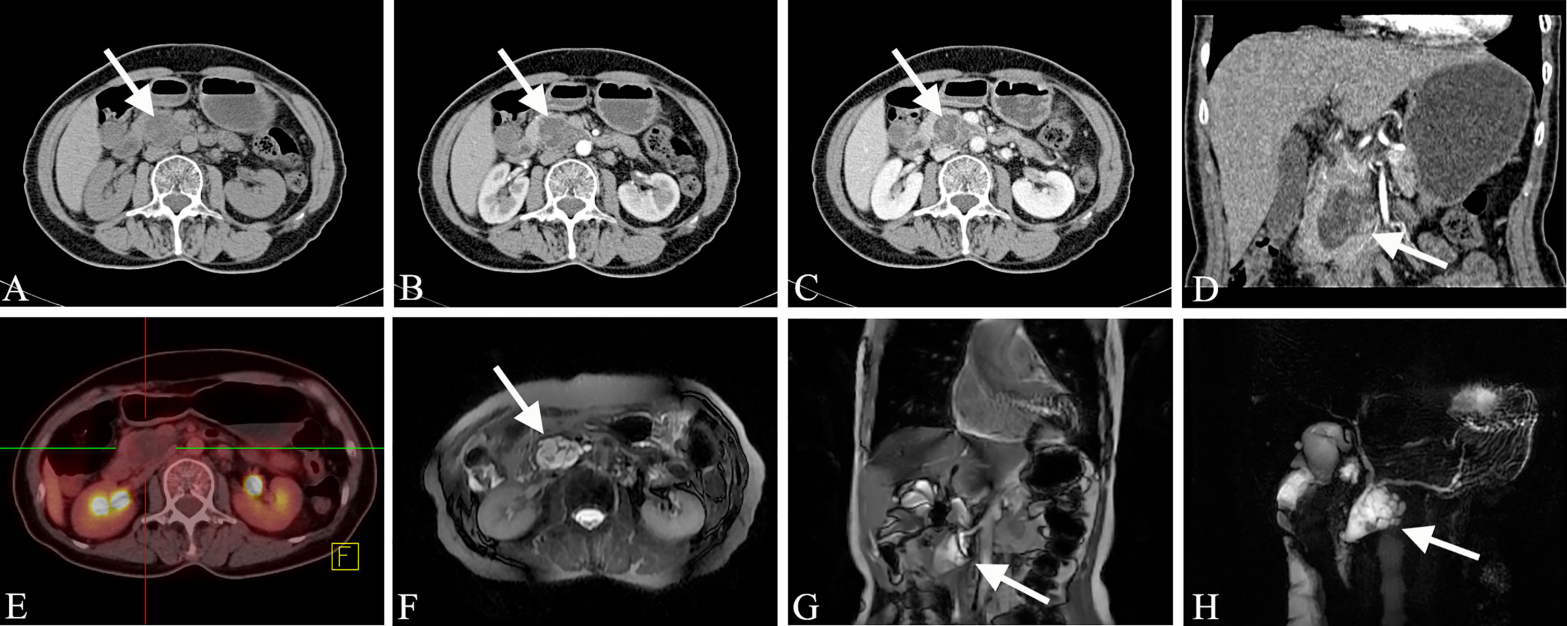
Mixed-type IPMN (Female, 56y, abdomen pain for half year). **(A–C)** The CT plain scan, arterial phase and venous phase at the same level; **(D)** Coronal image in arterial phase. **(E)** PET cross-sectional image; **(F)** T_2_-weighted cross-sectional image; **(G)** T_2_-weighted coronal image; **(H)** MRCP reconstruction map; **(A)** The CT plain scan showed multilocular cystic mass of pancreatic head with septation and clear boundary. The density is slightly higher than that of water. **(B, C)** Contrast enhanced scan showed moderate enhancement of mural nodules in the dilated MPD and branch pancreatic duct. **(D)** Diffuse dilatation of the MPD with enhanced mural nodules, which is the key to the diagnosis of mixed-type IPMN. **(E)** No obvious FDG uptake was found in the lesions by PET-CT. **(F–H)** Magnetic resonance imaging showed a multilocular cystic mass in the pancreatic head with multiple mural nodules, which communicated with the pancreatic duct. The white arrows in the Figures only indicate the location of the neoplasm.

Mixed-type IPMN often comprises both branched IPMN and MPD IPMN, but it is not a simple combination of the two. In MT-IPMN, the expansion of the MPD can be localized or diffuse, and localized expansion can manifest as multiple discrete segmental expansions, but no beaded changes occur. There is no strict boundary between the limited MPD dilatation and branched IPMN with mild MPD dilatation. Some studies believe that mixed IPMN is caused by the further development of branched IPMN, which is the key to distinguishing between branched IPMN and the mixed type. It is unclear whether there are tiny nodules in the MPD that are adjacent to the expansion, and if there are tiny nodules in the expanded MPD, it is of a mixed-type (**Figure 4**). Mixed-type IPMN can be accompanied by one or more branch types. Therefore, the imaging manifestations of mixed-type IPMN are more complex and can differ, but simultaneous expansion of the BPT and MPD is also the easiest subtype to diagnose.

Magnetic resonance T_1_WI showed that the liquid in the pancreatic duct of the IPMN dilated pancreatic duct had a low signal that was slightly higher than water; T_2_WI showed a high signal that was slightly lower than water. Some lesions showed hyperintensity on T_1_WI and hyperintensity on T_2_WI. The spatial resolution of MRI is limited, and the ability to show small mural nodules is not as good as CT, but MR helps to show larger nodules. The nodules show a lower signal on T_1_WI, which is between normal pancreatic tissue and dilated pancreatic duct fluid. T_2_WI is helpful to show streak-like hypointense separation or dilated pancreatic duct wall within the hyperintensity of branched IPMN, showing isohypointense mural nodules in the dilated pancreatic duct. The DWI signal of pancreatic IPMN varies greatly. Some DWI is iso-intense, and some is hyperintense. DWI helps to detect metastatic lymph nodes. MRCP helps to determine the relationship between the lesion and the MPD and shows the MPD protruding into the duodenum. Thin-layer MRCP is more helpful to determine the relationship between the MPD and the duodenum. During dynamic enhancement, the enhancement of the pancreatic duct wall of IPMN is similar to that of CT, the enhancement of small nodules is not as good as that of CT, and the enhancement of large nodules is close to or better than CT.

### The CT and MR Manifestations of MCN

Mucinous cystadenoma with invasive carcinoma often manifests as multilocular cystic lesions with uneven wall thickness, wall nodules and calcifications in the lesion. After enhancement, the intracapsular septum thickens, and the wall nodules are obviously strengthened. Sometimes it is not easy to distinguish between benign and malignant tumors by imaging. If the following signs appear in the cyst, mucinous cystadenoma with invasive carcinoma is often indicated: ①There are more solid components in the cyst. ②There are obviously enhanced mural nodules. ③ Irregular thickening of the cyst wall and the presence of multiple daughter cyst near the large cyst. ④Local pancreatic lymphadenopathy and intrahepatic metastasis are observed and adjacent large blood vessels have been invaded. ⑤The tumor is large, with a diameter >8 cm.

**Figure 5:**
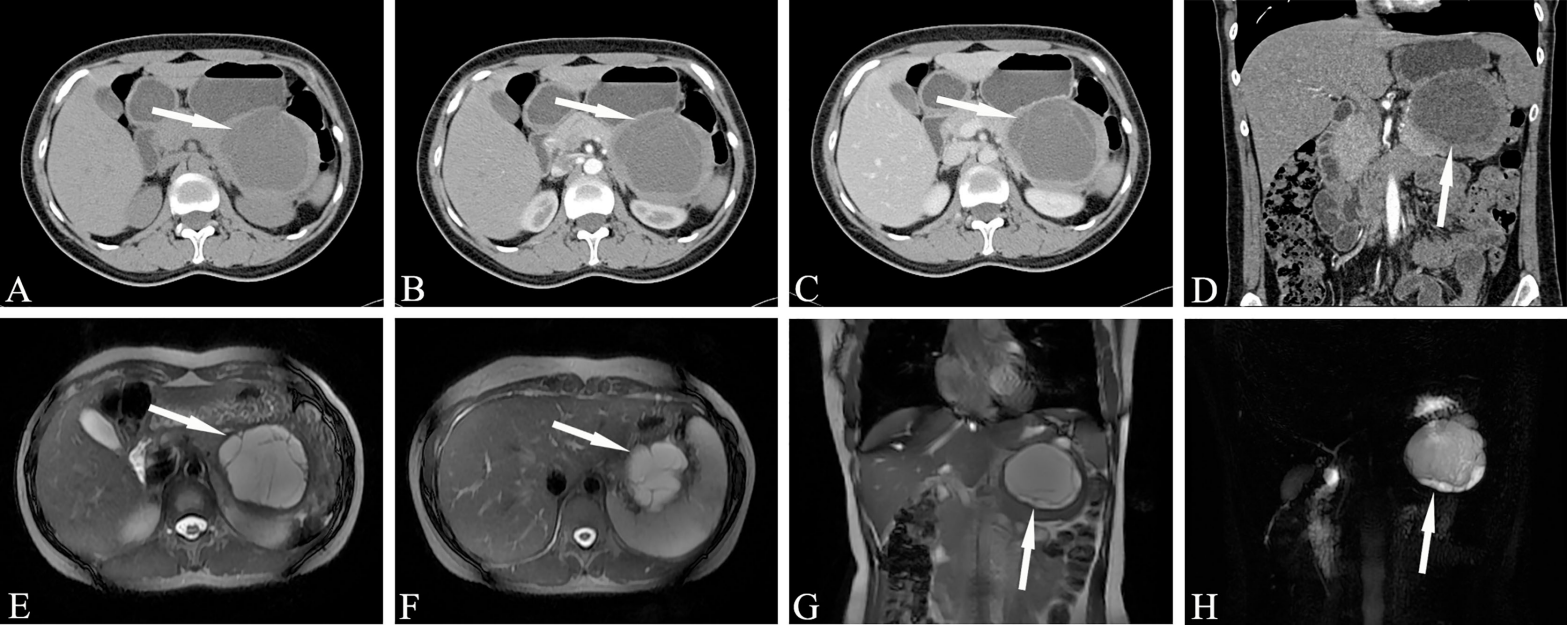
Mucinous cystic neoplasms, MCN (Female, 33y, physical examination revealed a pancreatic mass for one month). The sequence distribution of images is the same as that in **Figure 2**. **(A–D)** Huge cystic mass in the body and tail of the pancreas, irregular in shape, high tension, watery low density, thin and uniform cyst wall, multiple thin septum and small walled cysts can be seen in the cyst, and the septation and mild cyst wall can be seen enhanced after contrast injection. **(E–H)** Magnetic resonance images showed clearer septum and small sacs. There were no signs of pancreatic duct dilatation. The white arrows in the Figures only indicate the location of the neoplasm.

Mucinous cystadenoma usually manifests as a clear boundary with hypo-intensity on T_1_WI and hyper-intensity on T_2_WI, but sometimes it has different manifestations due to the composition of the cyst fluid. The advantage of MRI is that it can accurately reflect the composition of the cyst fluid of mucinous cystadenoma. Sometimes the signal on T_1_WI is uneven and has a high signal, which is pathologically related to mucin in the cyst fluid or intracystic hemorrhage. In addition, MRI shows better separation between the wall and wall nodules in the lesion capsule than CT. The cyst cavity of mucinous cystadenoma is generally not connected to the pancreatic duct, which helps to distinguish it from intraductal papilloma (communicating with the pancreatic duct). MRCP examination is helpful for determining whether the mucinous cystadenoma is connected to the pancreatic duct. MR examination of mucinous cystadenoma with invasive carcinoma can not only show that the tumor is cystic but also clearly depicts the tumor cyst wall, septum and mural nodules (**Figure 5**).

Uneven thickening of the intratumoral septum and cyst wall or the appearance of mural nodules, invasion of the common bile duct (CBD) or pancreatic duct (PD) and surrounding blood vessels are all helpful for the diagnosis of mucinous cystadenoma with invasive carcinoma. MR examination is helpful for the differentiation of benign and malignant pancreatic mucinous cystadenoma. Since the blood supply of the tumor is mainly concentrated in the cyst wall and septum, enhancement may appear after an enhanced scan.

### The CT and MR Manifestations of SCN

**Figure 6:**
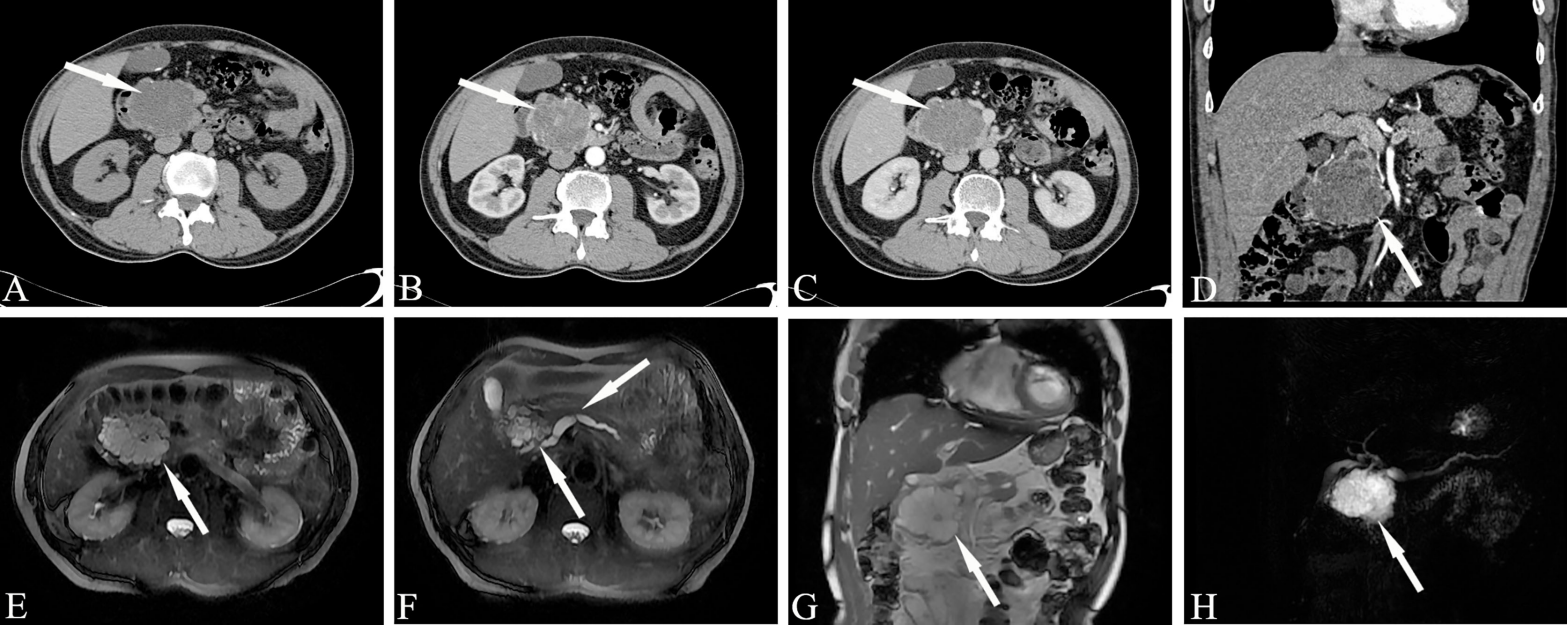
Serous microcystic adenoma, SMA (Male, 65y, physical examination revealed a pancreatic mass for one month). The sequence distribution of images is the same as that in **Figure 2**. **(A–D)** Polycystic or honeycomb cystic foci in the head of the pancreas, with a lobulated outline, like a collapsed wall dumping to the center, and slightly continuous enhancement of the cyst wall and septum. Punctate calcification can be seen in the capsule wall. The enhanced scan shows progressive medium-strength enhancement of the central scars. **(E–H)** Magnetic resonance imaging reveals microcapsule-like structures more clearly and the MPD was slightly dilated. A stellate scar can be seen in the center of the lesion. The white arrows in the Figures only indicate the location of the neoplasm.

CT scans of serous microcystic adenoma show a clear boundary, lobulation, and a mass formed by several small water-like cysts. The diameter of a single capsule is usually less than 2 cm. Star-shaped central scars can be seen in the lesions, and calcifications usually occur in the central scars. The enhanced scan shows progressive medium-strength enhancement of the central scars. It is often difficult to identify the tumor septum when it is thin, and enhancement can help with visualization when it is thicker. Star-shaped central scarring with or without calcification is considered to be a specific manifestation of serous microcystic adenoma. CT showing the intratumoral septum, central scar and size of the cyst is key to the imaging diagnosis of microserous microcystic adenoma. Sometimes serous microcystic adenomas are composed of numerous tiny vesicles, which show a honeycomb or spongy appearance, and most of the single cysts are one to several millimeters long (**Figure 6**). Clear edges and a cystic space with soft tissue structure can be seen on plain CT scan, and moderate enhancement is observed during the enhanced scan. At this time, it should be distinguished from SPN. When the lesion wall is thick, the internal components are complicated, and there is bleeding, calcification, liquefaction and necrosis, a solid pseudopapillary tumor is indicated.

MR shows serous cystadenoma basically similar to the CT appearance. However, the ability of MR to display calcification is weaker than that of CT, and MR can better display intracapsular hemorrhage, separation, and capsule wall. it can reveals more soft tissue information than CT scans. Soft tissue display can provide more information than CT. In addition, the display of the pancreatic duct and bile duct is more valuable for the differential diagnosis of diseases. Serous microcystic adenoma showed typical polycystic or honeycomb changes on MRI. The contents of the sac are a clear protein-containing liquid, generally showing a liquid signal shadow of long T_1_ and long T_2_. Sometimes the cyst cavity shows a slightly low signal shadow on T_2_WI, which is caused by the local fluid concentration in the cyst cavity and the high protein content. Small cysts can only sometimes be a few millimeters. Intensified scanning of the cyst wall and separation are often mildly continuously enhanced. The lobulated contour, as if the collapsed wall dumped toward the center, is a feature of microcystic serous cystadenoma caused by the traction of the central star-shaped scar; the central scar sometimes has calcification, which appears to be sunlight-radiating on CT, with certain characteristics, but MRI has obvious shortcomings in showing scar tissue calcification.

MRI can almost show the cyst wall and the space within it, even for tumors with small diameters. The intratumoral septum can be seen more clearly on T_2_WI. Serous tumor microcystic adenoma of the pancreas generally does not cause dilation of the CBD and PD. Although serous microcystic adenoma of the pancreatic head is adjacent to the CBD, there are few signs of abnormal compression or obstruction in the bile duct system. This may be due to the tumor’s soft body and slow growth, which does not compress the bile duct, also proving that the tumor is not aggressive. However, a small number of cases of serous microcystic adenoma with mild dilation of the pancreatic duct or CBD have also been reported. Mild pancreatic duct widening may be caused by mild compression changes or mild inflammatory changes in the pancreas. The relationship between the cyst cavity and the pancreaticobiliary duct is of great significance to the diagnosis and differential diagnosis of this disease.

**Figure 7:**
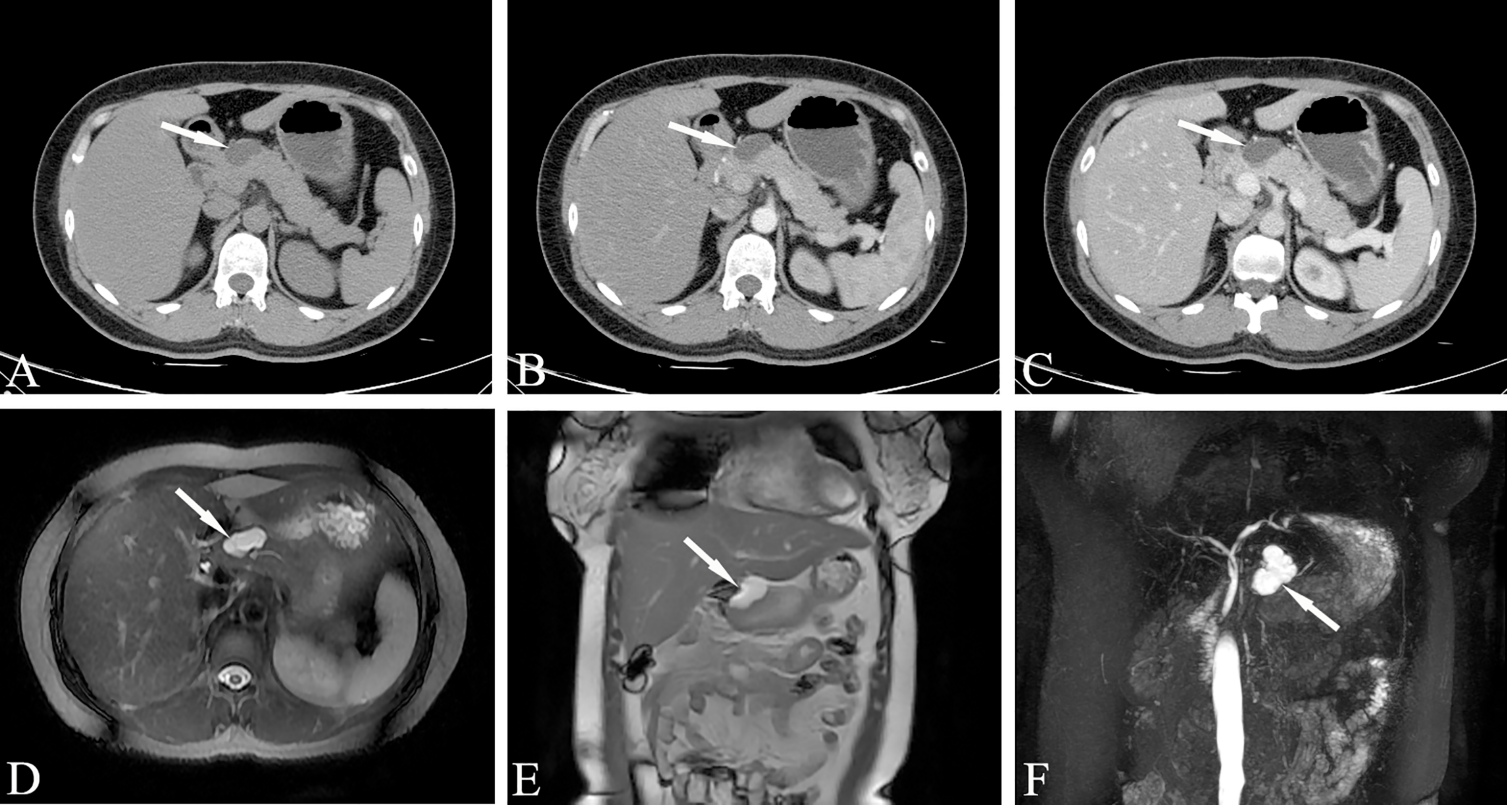
Serous oligocystic adenoma, SOA (Female, 39y, physical examination revealed a pancreatic mass for two weeks). **(A–C)** The CT plain scan, arterial phase and venous phase at the same level; **(D)** T_2_-weighted cross-sectional image; **(E)**. T_2_-weighted coronal image; **(F)** MRCP reconstruction map; **(A–C)** Low-density cystic mass in the neck of the pancreas with clear boundary and uniform density, no enhancement on dynamic contrast enhancement phase. **(D–F)** Magnetic resonance fat suppression T_2_WI showed a small cyst and a thin-walled separation next to the large cyst, and no signs of pancreatic duct dilatation. The white arrows in the Figures only indicate the location of the neoplasm.

Serous microcystic adenomas caused by pancreatic tail duct dilatation should be differentiated from BD-IPMN. MRCP can easily determine the relationship between the neoplasm and the pancreatic duct. Microcystic cystadenoma with typical changes and other pancreatic cystic tumors are not difficult to distinguish. Serous oligocystic adenoma tumors show typical unicystic or multicystic changes on MRI. Cystic lesions are larger than serous microcystic adenomas. The lesions are clearly separated from the surrounding pancreas, and the edges are smooth. The characteristics of the signal of the cyst contents and the relationship with the pancreaticobiliary duct are roughly similar to those of serous microcystic adenoma. There is no sign of communication between the cyst cavity of the lesion and the pancreatic duct, and the adjacent CBD often shows no obvious compression or obstruction (**Figure 7**).

**Figure 8:**
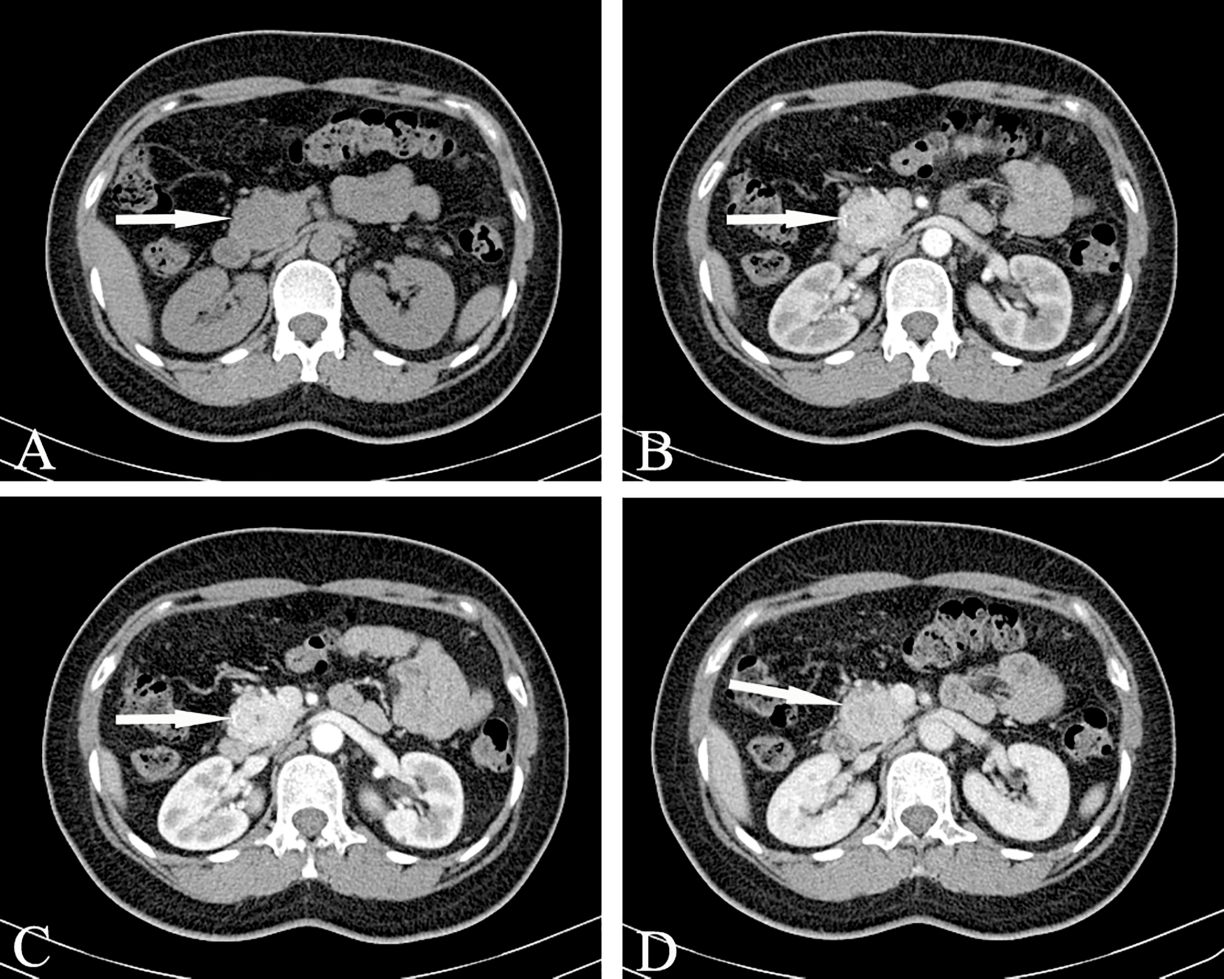
Solid serous cystadenomas, SSCA (Female, 43y, physical examination revealed a pancreatic mass for three weeks). **(A–D)** The CT plain scan, arterial phase, portal and venous phase at the same level; **(A)** The CT plain scan showed a solid mass in the head of the pancreas, with a CT value of about 31HU. **(B)** In the arterial phase, the mass of the pancreatic head was obviously enhanced, with a CT value of about 123HU. The focal low enhancement area can be seen in the center. **(C)** In the portal phase, the neoplasm showed progressive significantly enhancement, at this time, the CT value was 163HU; **(D)** The contrast wash-out can be seen in the venous phase of the mass, with a CT value of about 120HU. The overall manifestation was solid tumor with rich blood supply of pancreas. The white arrows in the Figures only indicate the location of the neoplasm.

Solid serous cystadenoma contains a large number of fibrous interstitial blood vessels, and there is no cyst in the tumor according to general pathology. Only tiny cysts can be seen under the microscope, with abundant interstitial blood vessels (**Figure 8**). This type of cystadenoma is rare. Because the capsule is very small, it is difficult to display watery signals in the lesion on T_2_WI, which often leads to the misdiagnosis of pancreatic neuroendocrine tumors.

### CT and MR Manifestations of SPN

**Figure 9:**
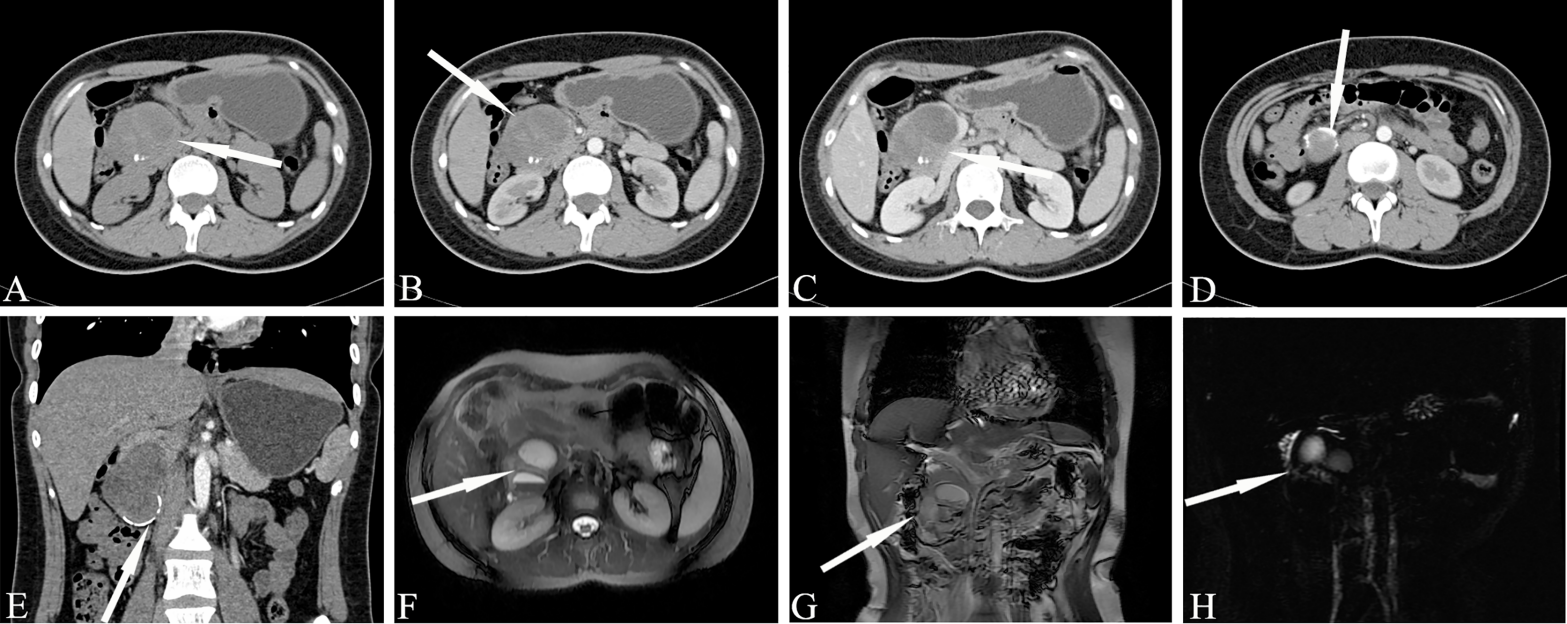
Solid pseudopapillary neoplasm, SPN (Female, 28y, physical examination revealed a pancreatic mass for a week). **(A–C)** The CT plain scan, arterial phase and venous phase at the same level; **(D)** Coronal image in arterial phase. **(E)** Arterial phase cross-sectional image at another level of the same patient. **(F)**. T_2_-weighted cross-sectional image; **(G)** T_2_-weighted coronal image; **(H)**. MRCP reconstruction map; **(A)** The CT plain scan showed a low-density mass in the head of the pancreas with cystic degeneration. Calcification was visible in the mass. **(B–E)** The solid component reinforcement was not obvious. The incomplete arc-shaped calcification of the envelope can be seen. **(F–H)** MRI shows old hemorrhagic signal with fluid-fluid level. There were no signs of pancreatic duct dilatation. The white arrows in the Figures only indicate the location of the neoplasm.

Regarding the typical CT appearance of a SPN, the solid part of the pancreas is slightly low-density, and cystic necrosis is shown as a lower-density area. The pathological basis that causes uneven density and mixed signals is tumor cystic transformation, hemorrhage, and the calcification of lesions(**Figure 9**). The distribution of cystic and solid components are also different; they can exist alternately, solid components can be located around the tumor, or multiple cysts of different sizes can be located at the edge of the tumor. Pathologically, the tumor cells in the pseudopapillary area form branched pseudopapillae with slender fibrous blood vessels as the axis. The cells are arranged in nests or lumps and in multiple layers. They are far away from the tumor cells around the blood vessels and are prone to degeneration. Necrosis, liquefaction and cystic changes can occur. Histologically, bleeding is prone to occur due to the large number of fragile, thin-walled blood vessels and the lack of a strong stent structure. Hemorrhage is one of the characteristics of this tumor.

**Figure 10:**
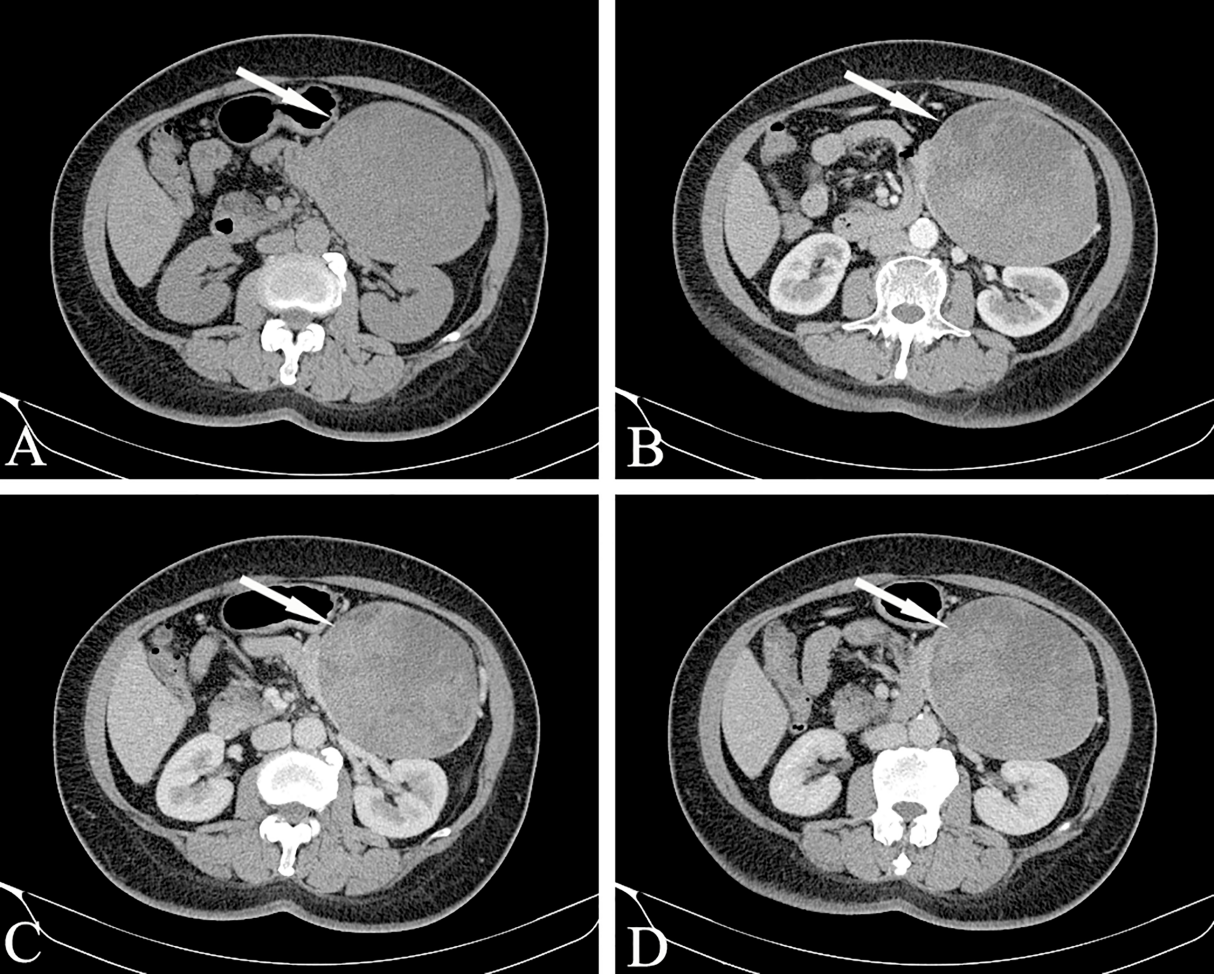
Solid pseudopapillary neoplasm, SPN (Female, 58y, physical examination revealed a pancreatic mass for one month). **(A–D)** The CT plain scan, arterial phase, portal phase and venous phase at the same level. The lesions are large in size, with cystic and solid components visible inside. The cystic part is not enhanced, but the internal solid components are enhanced and distributed in patches in low-density liquid tissue, showing the “floating cloud sign”. Besides, note that the pancreatic tissue had a “cup-mouth” boundary. The white arrows in the Figures only indicate the location of the neoplasm.

Bleeding can occur in the cystic part or the solid part, showing gel-like or cystic tissue; the cystic and solid components of CT are scattered and patchy and show a high density. Calcification in the lesion is more common and can manifest in various ways: small spots, diffuse calcification, incomplete arc-shaped calcification of the envelope (**Figure 9**), and sometimes complete arc-shaped calcification. If the lesion is mainly cystic, most of the cyst is not strengthened, and a few solid parts inside are obviously strengthened, which are distributed in the low-density liquid tissue in the form of sheets, forming the so-called “floating cloud sign” (**Figure 10**). The surrounding envelope is obviously enhanced. In the case of a cystic solid structure, the solid part of the arterial phase is mostly papillary or wall nodular enhancement. For the solid structure, the solid part of the arterial phase is slightly enhanced, and the parenchymal and delayed phases are further strengthened, showing progressive filling, but both are lower than the degree of pancreatic parenchymal enhancement.

For larger lesions, where cystic and solid lesions are often the main focus, the cystic component is not enhanced after enhancement, and the solid and cystic structures are clearly demarcated. Note that the pancreatic tissue has a “cup-mouth” boundary (**Figure 10**). Although the lesion is sometimes large, the MPD or CBD is generally not dilated. In a few cases, it may be slightly dilated, usually due to tumor compression of the adjacent duct. MRCP shows expansion of the MPD more intuitively than MDCT. A larger SPT can cause compression of adjacent blood vessels in the portal vein, splenic vein, inferior vena cava, and renal vein.

The MRI scan of SPN showed tumors with mixed signals on T_1_WI and T_2_WI and slightly hyper-intensity on DWI. The basis for the confounding of tumor signals is the tumor’s cystic degeneration, necrosis, hemorrhage and calcification. MRI is more sensitive to the detection of tumor hemorrhage than CT. Usually, hemorrhage MRI shows a hyper-intensity on T_1_WI and a hyper- or hypo-intensity on T_2_WI. Due to the coexistence of blood and other liquid components, signs of stratification can be seen, showing liquid levels. MRCP helps to show dilatation of the pancreaticobiliary duct.

### Endoscopic Ultrasound With Fine-Needle Aspiration

Recently, EUS has been recognized as an essential diagnostic tool for PCN management. When the results of the radiological diagnosis of malignant tumors are certain and/or when the PCNs are considered to have clinical or radiological characteristics, EUS is nominated as a second-line examination method after CT/MRI. Because the stomach and pancreas are adjacent to each other in the body, the EUS transducer can be placed close to the pancreas, and the gland can be clearly imaged. In this way, the pancreatic cyst wall and its contents can be evaluated in detail, and internal septae and solid areas within the cysts can be differentiated.

One study (77) showed that EUS is the best diagnostic method for differentiating nonneoplastic cysts and PCNs and to characterize PCNs, being superior to both CT and MRI. However, another large multicenter study reported the opposite result: the accuracy of applying EUS to diagnose mucinous versus nonmucinous cysts was only 51% (16). It is speculated that one of the limitations of EUS may be due to different interpretations among endoscopists. Another study revealed that the accuracy of detecting neoplasms with malignant potential ranged from 40% to 93% among 8 different endoscopists invited to interpret the same EUS procedure (78).

Making an accurate diagnosis with cross-sectional imaging and EUS alone is challenging, so EUS-FNA (fine needle aspiration) is frequently employed to obtain a cyst aspirate. Cyst fluid cytology suffers from poor sensitivity, which is specific. During EUS, various analyses (cytology, biochemistry and molecular) of pancreatic cyst fluid acquired from FNA can observably increase the accuracy of diagnosis (1, 16, 79). In terms of the rate of correct diagnosis, EUS-FNA increased the accuracy by 36% after CT and the accuracy by 54% after MRI (80). The risk of infection, hemorrhage, and pancreatitis of EUS-FNA increases compared to noninvasive imaging, while most studies have shown that its benefits outweigh the risks (81, 82).

### Cytology

EUS-FNA is a commonly used method of diagnosing IPMNs. However, the interpretation of cytological features relies on clinical and radiological findings. The presence of large amounts of thick mucin in the correct clinical setting can only suggest a diagnosis of IPMN. In contrast, it is difficult to distinguish the presence of limited mucin and low-grade mucinous epithelium from the presence of normal gastric epithelium (4). Compared with low-grade IPMN, the following features are more supportive of differentiating HGD IPMN: background of necrosis, aberrant chromatin patterns (hypochromasia or hyperchromasia), the presence of large vacuole single cells, significant nuclear irregularities, increased nuclear-plasmic ratio, and small cell sizes (≤12 μm duodenal cell) (83). Furthermore, The high-grade atypia of IPMNs tend to be larger (≥30 mm), have enhanced mural nodules (≥5 mm) (84) or have solid contents and dilated MPD (≥5 mm) (85).

Differentiating between IPMN and MCN is also difficult for cytopathologists. Due to involvement with the pancreatic duct observed with IPMN but not MCN, correlation with radiologic findings can significantly facilitate diagnosis compared to others. At the same time, mucinous cystic tumors develop almost entirely in females, and presence of an ovarian stroma is pathognomonic. Aspirates of low-grade MCNs (mucinous cystic adenomas) that account for more than 75% of MCNs will show honeycomb sheets of bland mucin-containing epithelium but often lack the presence of complex papillary architecture compared to high-grade MCNs (4). The mucin-containing nucleus has a smooth contour, fine chromatin, and inconspicuous nucleoli (86).

EUS with FNA for SCNs has both low specificity and sensitivity, SCNs usually contain hemosiderin-laden macrophages and paucicellular cells with clear or hemorrhagic backgrounds. The highly vascularized fibrous septa of the SCN leads to the hemorrhagic nature of these specimens. SCNs do not involvement with the PD system and have low CEA levels, with cysts are often filled of clear-yellow serous fluid with low viscosity, compared with IPMNs. The cells of SCNs are bland. The nucleus is round, and the contour is smooth. Chromatin is evenly distributed in the nucleus, and the nucleoli are inconspicuous. When the background contains mucin, associate with CEA levels and radiologic imaging would be cautiously exclude MCN. Macroscopically, SCNs are usually arranged around star-shaped scars, which show cysts with a distinctive spongy or honeycomb appearance.

The contents of hypercellular smears shown by EUS-guided FNA of SPTs include slender papillary fragments with fibrous vascular stalks and perivascular myxoid matrix. They are arranged by monomorphic cubic cells into cohesive groups and isolated cells. The neoplastic cells are round to oval, and the cytoplasmic boundary was unclear. The nucleus is grooved or bean-shaped, while the chromatin is fine-grained, and occasionally invisible or small nucleoli can be seen. Macroscopically, SPNs are large, with round to oval shapes, clear margin and fibrous pseudocapsules. SPNs are complex neoplasms with varies components (e.g., solid, cystic, hemorrhagic, and necrotic). Cystic degeneration is a common phenomenon that occurs during progression. Moreover, the larger the neoplasm is, the more obvious the cystic component (45, 87).

### Tumor Markers

Studies have shown that pancreatic cyst fluid analysis of tumor markers and molecular markers can help characterize PCNs. At present, perfect biomarker testing for detecting pancreatic tumor has not yet been developed. The most commonly used blood test to monitor and detect pancreatic cancer is the serum marker CA 19-9. However, its sensitivity is limited, especially for small malignancies (88). When CEA levels > 192 ng/mL, the sensitivity and specificity of the diagnosis of mucus lesions are 73% and 84%, respectively (16). Negative and positive predictive values for mucin etiology are both 94% when CEA levels <5 ng/mL or >800 ng/mL (17). The utilization of CEA using pancreatic cyst fluid to diagnose malignant cysts is less effective, as a previous meta-analysis suggested that both the diagnostic sensitivity and the specificity were 63% (89). Chemical analysis of liquid CEA and amylase levels may be helpful, but this approach cannot differentiate between MCNs and IPMNs. Elevated CEA can be used as a marker to distinguish between mucinous and non-mucinous cysts rather than benign or malignant cysts. When the critical value of CEA was ≥192~200 ng/mL, the accuracy increased to 80% for the diagnosis of mucinous cysts, showing high specificity but low sensitivity (16).

The current view is that serous cystic adenomas originate from centroacinar cells, where staining for cytokeratins and calretinin is positive but staining for CEA, mucin, estrogen receptors, and progesterone receptors is negative. SCN and ductal adenocarcinoma and neuroendocrine tumors can be distinguished by inhibin and calcarein, which were found to be helpful immunostaining markers in recent studies (90, 91).

### Molecular Markers

To compensate for the limitations posed by cytology and tumor markers, specific molecular markers for diagnosing PCNs and predicting malignant tumors are currently being developed. A molecular DNA analysis method for pancreatic cyst fluid is currently on the market. However, a molecular analysis method for cyst fluid is still in development. KRAS mutations support the diagnosis of mucous cysts more accurately, but KRAS does not always indicate a malignant cyst. It may be helpful to use GNAS mutations to differentiate between obvious mucinous cysts and indolent cysts that can be managed conservatively (92). Genetic analysis showed that the dual mutations in KRAS and GNAS were highly specific for IPMN. Compared with MCNs and SCN (lack GNAS codon 201 mutations), several research found that mutations in GNAS codon 201 are present in some IPMNs (41%-66%) and can even reach 74% to 100% in enteric IPMN (18–20).

With the aim of distinguishing MCNs from other PCNs, such as IPMN and SPN, some research have revealed that MCN is a kind of cystic neoplasm without the GNAS mutation and generally without the CTNNB1 mutation (21). KRAS mutations have been reported in MCNs (50%-75%) (19, 21). In serous cystadenomas, the absence of CTNNB1 mutation can be used to distinguish them from SPNs. In addition, KRAS and GNAS mutations are often expressed in IPMNs and MCNs rather than SCNs. There are a few studies of protein expression in SCAs. VEGF is a protein inhibited by a kind of tumor suppressor that is usually encoded by the VHL gene. In cysts or pancreatic duct fluid, VEGF-A levels can aid in diagnosis. The sensitivity and specificity for diagnosing SCAs were 100% and 97%, respectively, when VEGF-A levels were > 8500 pg/mL and 100% and 90%, respectively, when VEGF-C levels were > 200 pg/mL (22). Combining both VEGF-A and VEGF-C provides 100% sensitivity and specificity for the diagnosis of SCA. In addition, VEGF-A (using a critical value of >5000 pg/mL) combined with CEA (<10 ng/mL) can detect SCAs with a sensitivity of 95.5% and a specificity of 100% (93).

Few research have focused on the glycoproteomics of SCAs. The research have shown that SCAs express MUC1 and MUC6 instead of MUC5AC, which provides proof that SCAs originate from pancreatic central cells and intralebar ducts (23, 24). An extracellular matrix protein implicated in pancreatic cancer called periostin was found to increase 8-fold in SCA cyst fluid compared to mucinous lesions (94). On the other side of the shield, serous cystadenomas are related to von Hippel Lindau (VHL) syndrome, while mutations in the VHL gene are present in all SCAs in patients with VHL syndrome. VHL loss-of-function mutations may also be reflected in the development of sporadic SCAs (95). The macrocyst (oligocyst) variant is a rare type of SCN with fewer but more numerous cysts and without a stellate central scar (96); solid variant, which is devoid of cysts; and mixed serous-neuroendocrine variant (91).

Studies have been performed to study several proteins related to the above genes in SPN tissue: B-catenin, androgen receptor, lymphoid enhancer-binding factor 1 (LEF1), and transcription factor for immunoglobulin heavy-chain enhancer 3 (TFE3) (25). Among them, B-catenin has a sensitivity of 98.9% and a specificity of 97% for the diagnosis of SPT, which is the most sensitive indicator of diagnosis. The combination of LEF1 and TFE3S increases the sensitivity to 100% but decreases the specificity to 91.9%. Another investigation explored the use of B-catenin to diagnose SPT, reporting a 100% sensitivity and 87% specificity (26). The combined application of B-catenin, TEF3, and SOX11 can be used to distinguish SPN, with a sensitivity and specificity of 97%. These tissue findings are also relevant for EUS-FNA biopsy samples (27). The absence of KRAS, GNAS, or RNF43 can distinguish SPTs from other PCNs. Because of the good prognosis of SPNs, complete resection of these inert neoplasms can be cured.

## The Management of PCNs

Because of the significant overlap in the morphology of benign and premalignant lesions, characterizing and managing PCN poses a substantial dilemma for the clinical arena. However, compared with clinical and radiological suspicion, the patients are the most important parameter leading to clinical decision-making in surgery treatment. Patients fitness for surgery are continuous variable that should be considered in terms of age, life expectancy, health status, degree of frailty, patient preference, motivation for surgery, and availability of benefit. This parameter is crucial because the overall malignancy rate of PCNs is low. Each patient should be carefully evaluated by clinicians according to the patient’s own situation after adequate consultation. Another significant factor in the final decision is the surgery type, as pancreaticoduodenectomy and distal pancreatectomy have different responsibilities in terms of morbidity, mortality, and sequelae.

There are two aspects that should guide the management of IPMN (1): whether the IPMN is malignant and (2) whether the IPMN will become malignant during a patient’s lifetime. Clinicians still face the problem of detecting the presence of a malignancy in IPMN and determining its future malignant potential (97). According to the 2012 international consensus guidelines (44), surveillance of BD-type IPMN without “high-risk stigmata” was recommended based on the size stratification. On the basis of American Gastroenterological Association Institute guidelines, patients with pancreatic cysts <3 cm without a solid component or a dilated pancreatic duct should undergo MRI at 1 year. If there is no change in size or characteristics, they should undergo MRI every 2 years afterward for a total of 5 years (70).

In the last 20 years, management recommendations for patients with IPMN have changed dramatically along with advances in our knowing of the natural history of this neoplasm. The reason for this evolution is that various studies have identified clinical, imaging and biologic predictors that may correctly distinguish IPMN with HGD and IC. Models with remarkable accuracy are being developed by combining clinical and imaging characteristics with promising cyst fluid markers. Given the relative rarity of this disease, enhancing constant international collaboration is necessary to successfully obtain a prevention strategy to reduce the incidence of pancreatic cancer arising from IPMN. Patients who have suspected findings but without absolute indications for surgery should undergo CE-EUS. For patients in whom it has been difficult to confirm malignancy under endoscopy, further development of the disease should be closely monitored by MRI/MRCP, tumor markers, and CE-EUS. Once an SCN is detected, then the focus should be excision and long-term monitoring based on questions surrounding symptoms of local growth and progression, not cancer development.

If patients are doubted of having IPMNS, MCNs, and SPTs, appropriate lymphadenectomy and negative resection margins based on intraoperative frozen section assessment should be considered during surgical resection to completely remove the tumor. Given that the disease is usually malignant, parenchyma-sparing pancreatectomy is not a safe procedure for whole PCN cases. Overall, it should be considered only for selected cases or for SCNs.

However, it has not been demonstrated that prolonged follow-up reduces cancer-related mortality, but all these studies revealed that cyst stability over 5y does not exclude the risk of future progression to pancreatic cancer, and thus, there is a lifelong risk of malignancy. Therefore, follow-up should be continued due to the importance of repeated observations for risk stratification. Currently, several follow-up schedules have been suggested in the current guidelines (92, 98); unfortunately, none of these schedules have been shown to be highly cost-effective. In general, the authors recommend that MRI/MRCP and oncological markers should be followed-up every 6 months for the 1st year in the absence of the suspicious features mentioned above. In the absence of progression, it is necessary to maintain follow-up with MRI/MRCP and serum markers for 12 or 18 months.

## Conclusions

In recent years, the incidence of PCLs, especially PCNs, has increased daily. Due to the particularity of its anatomical location, the complexity of endocrine function, the diversity of pathological types, and the unsatisfactory prognosis, clinicians have become a great concern. The key issue is early diagnosis and early treatment, so the imaging diagnosis of pancreatic cystic tumors shows important value in diagnosis. The accuracy of preoperative imaging diagnosis is essential to improve clinicians’ confidence in surgery and individualized management. In conclusion, we hope that in the future, imaging biomarkers can be used along with histopathology to provide greater theoretical support for the precise treatment of tumor patients.

## Author Contributions

FH and YH are the co-first authors of this paper and have contributed equally to this work. WP and TT are the co-corresponding authors. They are responsible for the quality of the review. All authors made substantial contributions to all of the following: (1) the conception and design of the study, or acquisition of data, or analysis and interpretation of data, (2) drafting the article or revising it critically for important intellectual content, (3) final approval of the version to be submitted. Publication is approved by all authors. There are no conflicts of interests associated with this work.

## Funding

The research was supported by the National Natural Science Foundation of China (No. 81971687/No. 81801702)

## Conflict of Interest

Author PW was employed by GE Healthcare.

The remaining authors declare that the research was conducted in the absence of any commercial or financial relationships that could be construed as a potential conflict of interest.

## Publisher’s Note

All claims expressed in this article are solely those of the authors and do not necessarily represent those of their affiliated organizations, or those of the publisher, the editors and the reviewers. Any product that may be evaluated in this article, or claim that may be made by its manufacturer, is not guaranteed or endorsed by the publisher.
